# 964. Real-world dalbavancin use for serious gram-positive bacterial infections: comparing outcomes between people who use and do not use drugs

**DOI:** 10.1093/ofid/ofad500.025

**Published:** 2023-11-27

**Authors:** Sarah Zambrano, Molly L Paras, Joji Suzuki, Jeffrey C Pearson, Harry Schrager, Jason Mallada, Brandon Dionne, Katie Fairbank-Haynes, Marlene Kalter, Veronica Szpak, Sara Prostko, Daniel A Solomon

**Affiliations:** Brigham and Women's Hospital, Jamaica Plain, MA; Massachusetts General Hospital, Harvard Medical School , Boston, MA; Brigham and Women's Hospital / Harvard Medical School, Boston, MA; Brigham and Women's Hospital, Jamaica Plain, MA; Tufts Medical School/Newton Wellesley Hospital, Newton, Massachusetts; Newton-Wellesley Hospital, Newton, Massachusetts; Brigham and Women's Hospital, Jamaica Plain, MA; Newton Wellsley Hospital, Newton, Massachusetts; Newton-Wellesley Hospital, Newton, Massachusetts; Brigham and Women's Hospital, Jamaica Plain, MA; Brigham and Women's Hospital, Jamaica Plain, MA; Brigham and Women's Hospital / Harvard Medical School, Boston, MA

## Abstract

**Background:**

Dalbavancin has been used off label to treat invasive gram-positive bacterial infections due to its long half-life, eliminating the need for long term IV access and prolonged hospital stays. It is a particularly useful option in vulnerable populations such as people who use drugs (PWUD) who face unique challenges with long term IV antibiotics. This large, retrospective, multi-site study examined outcomes at 90 days in PWUD vs non-PWUD after treatment with dalbavancin for bacteremia, endocarditis, osteomyelitis, epidural abscess and septic joint infections.

**Methods:**

Patients at three university-affiliated hospitals who received dalbavancin for an invasive gram-positive infection between March 2016 and May 2022 were included for analysis. Characteristics of PWUD and non-PWUD were compared using chi-square for categorical variables, t-test for continuous variables, and non-parametric tests where appropriate. A logistic regression model was created where clinical cure at 90 days was assessed according to PWUD status with adjustments for age, housing status, and type of infection. For all analyses alpha was set to 0.05.

**Results:**

There were a total of 176 patients. Of those, 78 were PWUD and 98 were non-PWUD. PWUD were more likely to be younger (40.4 vs 56.2 years old; p< 0.0001), experiencing homelessness (24.4% vs 2%; p< 0.0001), and have a history of hepatitis C (65.4% vs 2%; p< 0.0001). Endocarditis, vertebral osteomyelitis, and epidural abscesses were more common infections among PWUD vs non-PWUD (20.5% vs 5.1%, 9% vs 0, and 9% vs 1%, respectively; P< 0.05 for all). There was no significant difference in length of hospital stay between the two groups. PWUD were more likely to have a patient directed discharge (27.3% vs 3.1%; p< 0.0001) and be lost to follow up (21.8% vs 6.1%; p < 0.002). Survival (78.2% vs 81.6%) and cure (71.8% vs 75.5%) at 90 days were not different between PWUD and non-PWUD, respectively. When controlling for age and housing status, the adjusted OR for clinical cure at 90 days among PWUD was 0.78 (95% CI 0.34-1.80).

**Figure:**
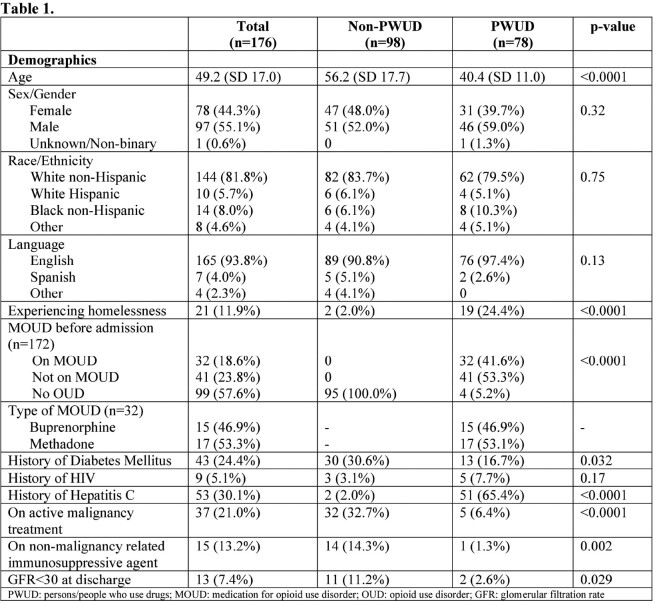

**Figure:**
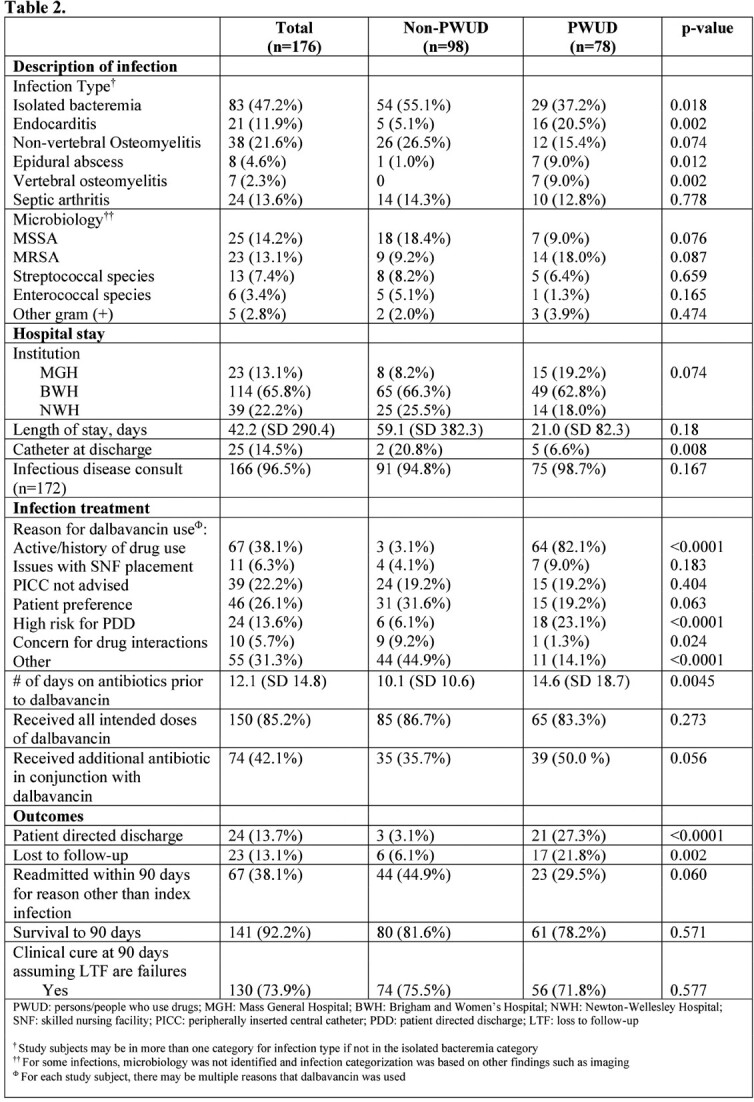

**Conclusion:**

Dalbavancin was an effective treatment option for invasive gram-positive infections in our patient population. Despite higher rates of patient directed discharge and loss to follow up, PWUD had similar rates of clinical cure at 90 days to non-PWUD.

**Disclosures:**

**Molly L. Paras, MD**, Angiodynamics: Honoraria|Angiodynamics: Honoraria **Joji Suzuki, MD**, Indivior: Grant/Research Support

